# Mining the relationship between COVID-19 sentiment and market performance

**DOI:** 10.1371/journal.pone.0306520

**Published:** 2024-07-05

**Authors:** Ziyuan Xia, Jeffrey Chen, Anchen Sun

**Affiliations:** 1 Antai College of Economics & Management, Shanghai Jiao Tong University, Shanghai, China; 2 Decision, Operations & Information Technologies, University of Maryland Robert H.Smith School of Business, College Park, MD, United States of America; 3 Department of Electrical and Computer Engineering, University of Miami, Coral Gables, Florida, United States of America; University of Basel, SWITZERLAND

## Abstract

In March 2020, the outbreak of COVID-19 precipitated one of the most significant stock market downturns in recent history. This paper explores the relationship between public sentiment related to COVID-19 and stock market fluctuations during the different phases of the pandemic. Utilizing natural language processing and sentiment analysis, we examine Twitter data for pandemic-related keywords to assess whether these sentiments can predict changes in stock market trends. Our analysis extends to additional datasets: one annotated by market experts to integrate professional financial sentiment with market dynamics, and another comprising long-term social media sentiment data to observe changes in public sentiment from the pandemic phase to the endemic phase. Our findings indicate a strong correlation between the sentiments expressed on social media and market volatility, particularly sentiments directly associated with stocks. These insights validate the effectiveness of our Sentiment(S)-LSTM model, which helps to understand the evolving dynamics between public sentiment and stock market trends from 2020 through 2023, as the situation shifts from pandemic to endemic and approaches new normalcy.

## 1 Introduction

Understanding and predicting the stock market behavior has always been a major goal for academia and industry, but an extremely difficult one. The efficient market hypothesis, which is an earlier theory of stock market prices by economist Eugene Fama, states that in an efficient market the current market prices is reflective upon all available and relevant information [1, 2]. Stock market price changes are driven by new, unpredictable information, making prices seem unpredictable. Incomplete public information leads to weaker market efficiency, where stock prices don’t accurately reflect true value. Reducing information asymmetry helps minimize market anomalies and volatility. By increasing the access to information and transparency to the public, strong market efficiency is achieved [3].

Strong market efficiency theoretically requires perfect information, but public access is often delayed or limited, as readily available data lags behind actual events. While news remains unpredictable, indicators derived from social media platforms can serve as asymmetric new information to aid in forecasting stock market performance. Therefore, this research aims to investigate how public sentiment on social media can gauge stock market performance, specifically by analyzing how COVID-19-related sentiment—concerning management, infection rates, and confidence in the economy and government—affects consumer investment behavior and stock market outcomes [4].

The reaction of the major global equity markets to COVID-19 in the early 2020 can be basically divided into the following parts in terms of timing [5].

- As of January 2020, infection was primarily confined to Asia, notably impacting the Hong Kong stock market, amidst escalating global concerns about the outbreak and its containment, which was not yet significantly reflected in global stock markets, showing only minimal fluctuations [6]. The World Health Organization declared the NCCP outbreak an “international public health emergency” on January 31, following the first reported fatal case in Wuhan on January 11, which confirmed human-to-human transmission, leading to widespread closures and increasing cases across Asia [7].
- In late February, a significant outbreak of Newcastle pneumonia in Europe, particularly after Lombardy’s partial lockdown on March 4, led to severe downturns in European and global stock markets, exacerbated by shocks from the nearing collapse of healthcare systems in Europe and the U.S., resulting in four U.S. market meltdowns by the end of March [8]. This turmoil saw the Dow Jones plummeting by 2,352 points on March 12 and an additional 2,997 points on March 17, marking historic drops and triggering global panic [9].

From January to Mid-March 2020, European and U.S. stocks fell by a deep while Asian markets fell by a relatively small amount. By April 2020, though infection rates have not improved [10], the stock market has been in steady recovery, indicating that the news of growing coronavirus cases was no longer negatively impacting the stock market [11, 12]. As of June 8, 2020, the WHO had announced that the COVID-19 situation is still worsening, but at the same time, the stock market was still growing for the fourth week in a row, with the S&P 500 returning to it’s position before the pandemic [13].

Since late 2020, the COVID-19 pandemic has profoundly influenced global markets, leading to a dynamic period characterized by both volatility and recovery. By 2021, extensive vaccination campaigns helped stabilize many economies, prompting a rebound in stock markets that had dipped during the initial stages of the pandemic. This recovery was periodically challenged by the emergence of new variants like Delta and Omicron, which introduced uncertainty and could trigger market fluctuations. However, by 2022 and 2023, as COVID-19 measures became more integrated into standard healthcare practices and economies adapted to new normals, the focus in financial markets shifted towards long-term growth strategies [14]. The introduction of booster shots and improved treatments helped maintain economic stability, reducing the immediate impact of subsequent waves of the virus on market performance. The ongoing adjustments in public health policy and vaccine distribution continued to play a crucial role in shaping economic forecasts and investor confidence, guiding the transition from pandemic crisis management to routine risk assessment in the financial sector [15, 16].

The pandemic’s economic impact and public opinion of governmental and institutional responses—encompassing supply chain, leadership, policy, and research—significantly influence consumer behavior in investing and spending [17]. Research using Google search data from the first four months of 2020 across 54 countries indicated varying severity and timing of consumer panic in response to movement restrictions, while other studies highlighted how policies like mandating face masks and lockdowns effectively curbed COVID-19 spread, significantly impacting market volatility and public perception, which in turn could be analyzed through natural language processing for text sentiment analysis [18].

We found Twitter to be an ideal source of capturing natural language public sentiment data, as it is a social media platform with over 300 million monthly active users [19]. Early 2010 research of understanding behavioral economics from a societal mood states indicates that it is possible to use text sentiment using deep learning models to correlate with the performance of the Dow Jones Industrial Average (DJIA) over time [20]. Later research found that in a short-window event study of a UK based political event, Twitter messages (tweets) were collected and filtered to labels pertaining to a political event. With later statistical forecast proving evidence of causation between the public sentiment, and the closing price with a slight time lag [21]. We further the research of using text sentiment analysis in order to assess whether public opinion representative of a consumer’s confidence in the COVID-19 situation could also be used to predict the stock market indexes [22].

To carry out our study, we developed a data pipeline capable of capturing live tweets via the Twitter API [23]. Leveraging the capabilities of social media platforms such as Twitter, we filtered tweets containing keywords indicative of pandemic relevance. These tweets were analyzed using a pre-trained Twitter sentiment model to assign sentiment scores, which were then normalized. The normalized scores were correlated with stock market indices and specific pandemic-related stocks, as obtained through the Yahoo Finance API [24], reflecting changes over time starting from late 2020. Additionally, we enriched our analysis by incorporating expert-labeled sentiment data from 2020 to 2023 to facilitate independent testing and validation. Our study employs the S-LSTM model to track the shifts in social media sentiment and its impact on market performance from the onset of the pandemic through to a phase of normalization. This approach underscores the viability of integrating sentiment analysis into financial market assessment, highlighting the utility of diverse data sources in understanding market dynamics during critical periods.

## 2 Related work

Predicting stock prices is nothing new. In the field of econometrics, many different methods have been applied to the prediction of stock prices. One of the famous model is Financial Time Series (FTS) [25]. FTS modeling has a long history, having first revolutionized algorithmic trading in the early 1970’s. FTS analysis consists of two types of analysis: fundamental and technical. However, both types of analysis have been challenged by the efficient market hypothesis (EMH) [26], a controversial hypothesis that has been around since 1970.

Since its introduction in 1970, the EMH has been controversial, assuming that stock prices are ultimately unpredictable. This does not limit the study to FTS modeling by using linear, nonlinear, and ML-based models. Because financial time series are non-stationary, non-linear, and noisy, it is difficult for traditional statistical models to predict them accurately. In recent years, more and more studies have attempted to apply deep learning to stock market forecasting, although it is still far from perfect.

In [27] propose a support vector machine (SVM) based stock prediction method to develop a two-part feature selection and prediction model, and demonstrate that the method has better generalization ability than traditional methods. In [28] propose a neural network for predicting stock prices using a feedforward multilayer perceptron with backpropagation of errors. The results show that the model is capable of predicting a typical stock market.

The research entered the LSTM era in 2017 and the proliferation of research using LSTM networks to process time series data. LSTM was proposed by [29] and recently refined and popularized by Alex Graves. [30] propose to add a time-weighted function to LSTM and the results outperform other models.

[31] It combines the LSTM and an attention mechanism to design an attention-based LSTM then compares it with the LSTM model, the LSTM model with wavelet denoising, and the gated recurrent unit(GRU) neural network model to show the advantages of the incorporation with the attention mechanism. Around the same time, a new architecture of neural network, Deep Wide Area Neural Network (DWNN), is proposed. The results show that the DWNN model can reduce the mean squared error of the forecast by 30% compared to the conventional RNN model. [32] proposed to integrate CNN and DWNN models into a single model, which can reduce the mean-squared error of forecasts by 30% compared to conventional RNN models. A hybrid neural network model is proposed for a quantitative stock selection strategy to determine stock market trends and then to predict stock prices using LSTM, and a hybrid neural network model is proposed for a quantitative timing strategy to increase profits. In [33, 34] use LSTM neural network, graph network and RNN to build models and find that LSTM can be better applied to stock prediction. In their paper [35] added investors’ sentiment propensity to the model analysis and introduced empirical modal decomposition (EMD) in combination with LSTM to obtain more accurate Stock Prediction. LSTM models based on attentional mechanisms are common in speech and image recognition, but are rarely used in finance.

Deep learning algorithms exhibit significant potential for prediction of the stock markets. For example, sentiment analysis can be applied to news, social media, etc. to analyze the overall sentiment of the market towards a particular stock or a particular sector [36–38]. The stock market is essentially a game process, and observing the game process through sentiment analysis is a better entry point for introducing existing analytic algorithms into the financial market. Stock prediction is never a matter of putting a simple time series data into deep learning and making money. There is a wide variety of data on stock buy points, trading volume, historical prices, etc., and they serve different purposes. Instead of trying to uncover the complex mathematical models of the stock financial market, we should change the entry point and analyze the relationship between stock price changes and emotional fluctuations from the perspective of the emotions of ordinary stockholders who buy stocks [39].

Researchers in recent years have tried to predict stocks using neural networks or other time series analysis techniques in financial market field. In particular, previous studies have demonstrated that algorithms like LSTM or other models can handle sequence data. Auto regressive integrated moving average (ARIMA) model leverages historical stock price data to identify patterns and trends, adjusting for noise and seasonality through differencing, autoregression, and moving averages to predict future prices by capturing the statistical relationships between past and future values within the time series data [40]. Temporal Convolutional Networks (TCNs), when combined with recurrent neural network layers and gated recurrent units in a hybrid deep neural network architecture, effectively predict chaotic time series by leveraging TCNs to extract low-level features and the recurrent layers to capture temporal information, outperforming other classical and deep learning models in terms of prediction accuracy as demonstrated by their superior root mean square error values [41]. Convolutional neural networks (CNNs) predict stock prices by processing historical market data through convolutional layers that identify spatial and temporal patterns, transforming these patterns into abstract features via activation and pooling layers, and then analyzing them with fully connected layers to produce predictive outputs, effectively focusing on significant signals amidst the noisy financial data [42]. Long Short-Term Memory (LSTM) models predict stock prices by effectively processing sequential historical market data through specialized memory cells that capture long-term dependencies while filtering out irrelevant information, enabling the network to identify complex patterns and relationships over time that are crucial for generating accurate forecasts in the highly volatile and non-linear environment of stock market analysis [43]. Overall, previous studies have highlighted that while models like LSTM, ARIMA, TCN, and CNN offer valuable insights into stock market forecasting by handling temporal patterns, statistical relationships, and feature extraction, their limitations in accounting for non-linear market complexities emphasize the need for innovative hybrid approaches that leverage each model’s strengths.

## 3 Data

In this section, we will describe how the data used in our study was obtained. By building a sentiment analysis tool, we obtained a fast, efficient and autonomous way to obtain real-time data and store it on a server. The Twitter data will help us to monitor the sentiment of Twitter users towards COVID-19 and can be generalized to any other topic after more experiments. In the stock market, we use Yahoo Finance’s API to obtain data on the US stock market. In addition to real-time streaming data we collect ourselves, we also incorporate other publish sentiment datasets from social media to evaluate our proposed framework.

### 3.1 Tweet API streaming data (2020)

#### 3.1.1 Data collection and sentiment analysis

We built a sentiment analysis tool that extracts COVID-19 related tweets every thirty minutes. A sentiment analysis model, trained on the Twitter sentiment dataset comprising Sentiment140 [44] and preprocessed-twitter-tweets [45], was deployed to assess the stored tweets and generate sentiment scores at thirty-minute intervals. The sentiment scores are defined in the range [−1, 0), 0, and (0, + 1] for negative sentiment, neutral sentiment, and positive sentiment.

We created a cluster on the Google Cloud Platform (GCP) [46] and built a Twitter text data streaming sentiment analysis tool with Flume, PySpark, and PyTorch. Twitter offers developer accounts, which we use to access Twitter’s API and set up the Flume Collecter on GCP cluster to collect streaming tweets. The keywords are shown in Table 1 set to fetch tweets from API.

**Table 1:** Keywords used to crawling COVID-19 tweets.

Epidemic						
covid-19	corona	virus	pandemic	mask	stay home	work from home
breathing	China	Wuhan	lock down	outbreak	testing site	asymptonmatic
quarantine	vaccine	CDC	N95	KN95	transmission	community spread
endemic	epidemic	flu shot	positive	sars-cov-2	isolation	
Panic-Buying						
toilet paper	pasta	rice	hoarding	fruit	vegetables	panic buying
supermarket	flour					

The tool reads the Tweets streaming from Hadoop Distributed File System (HDFS) first, which can scan the HDFS path and convert all readable text files in the path to RDD format. After we get the completed streaming data file, we need to clean the text file because Flume streams all texts given by Twitter API. After reviewing the raw data text file, we found that these texts were encoded by Unicode and contained different international languages. The texts also includes emojis, Apple Emoticon Package, href and HTTP links etc. The regular expression is the most popular way to clean text data. A regular expression, regex or regexp is a sequence of characters that define a search pattern. We also built a Unicode range check to filter out Chinese, Korean and Japanese. When people write a sentence in these languages, they will not include a space between every two words. The best way to split these sentences is by using a Nature Language Package to mark the sentences then split them. We simply split characters in Chinese, Korean and Japanese and keep other languages as the original.

After initial metadata cleansing, the tweets were stored on the server for further processing via a PySpark pipeline designed to analyze textual data from Twitter. The analytic process commenced with tokenization and the generation of n-grams to capture the contextual nuances within the text. Subsequently, these tokens were transformed into numerical vectors using CountVectorizer and adjusted via inverse document frequency (IDF), which emphasizes the significance of less frequent but potentially more impactful words. The resultant feature vectors were then inputted into a logistic regression classifier trained to discern sentiment labels from these features. This methodological framework not only quantifies tweet sentiment effectively but also facilitates systematic analysis and scoring. As described above, this Pyspark pipeline with our pre-trained PyTorch sentiment analysis model was employed to compute the sentiment scores of the tweets collected at thirty-minute intervals.

#### 3.1.2 Stock price

On the server, we ran the Yahoo Finance API and recorded the daily stock market data of the daily opening and closing prices, as well as trading volume for the three major stock indices (NASDAQ Composite, DJIA, and S&P 500). The stock market is not open on weekends and holidays, but still has trades occurring. Therefore we use Lagrangian interpolation to fill in all the missing data so that it corresponds to the daily sentiment analysis scores.

Financial time series data (especially stock prices) are subject to constant fluctuations due to seasonality, noise, and automatic corrections [47]. Traditional forecasting methods (FTS) use moving averages and differentials to reduce forecast noise. However, FTS is often unstable and there is overlap between useful signals and noise, which makes traditional denoising methods ineffective.

Wavelet analysis has made impressive achievements in the fields of image and signal processing. It can compensate for the shortcomings of Fourier analysis, and is therefore gradually being introduced into the economic and financial fields. The wavelet transform has a unique advantage in solving traditional time series analysis problems because it can decompose and reconstruct financial time series data from a wide range of time and frequency domains. The wavelet transformation essentially uses multi-scale features to denoise the data set, thus separating the useful signal from the noise efficiently [31] used the coif3 wavelet function for three decomposition layers and evaluated the effect of the wavelet transform by the signal-to-noise ratio (SNR) and root mean square error (RMSE). The higher the SNR, the smaller the RMSE, and the lower the denoising of the wavelet transform, the better the results. By using Eq (1).

$$\text{SNR}=10\times \text{log}[\frac{\sum_{j=1}^{N}x_{j}^{2}}{\sum_{j=1}^{N}{\left(x_{j}-{\hat{x}}_{j}\right)}^{2}}]$$ (1)

we can denoise the collected stock data, we can get the cleaned data for our method.

#### 3.1.3 Normalization and comparison

**Fig 1:**
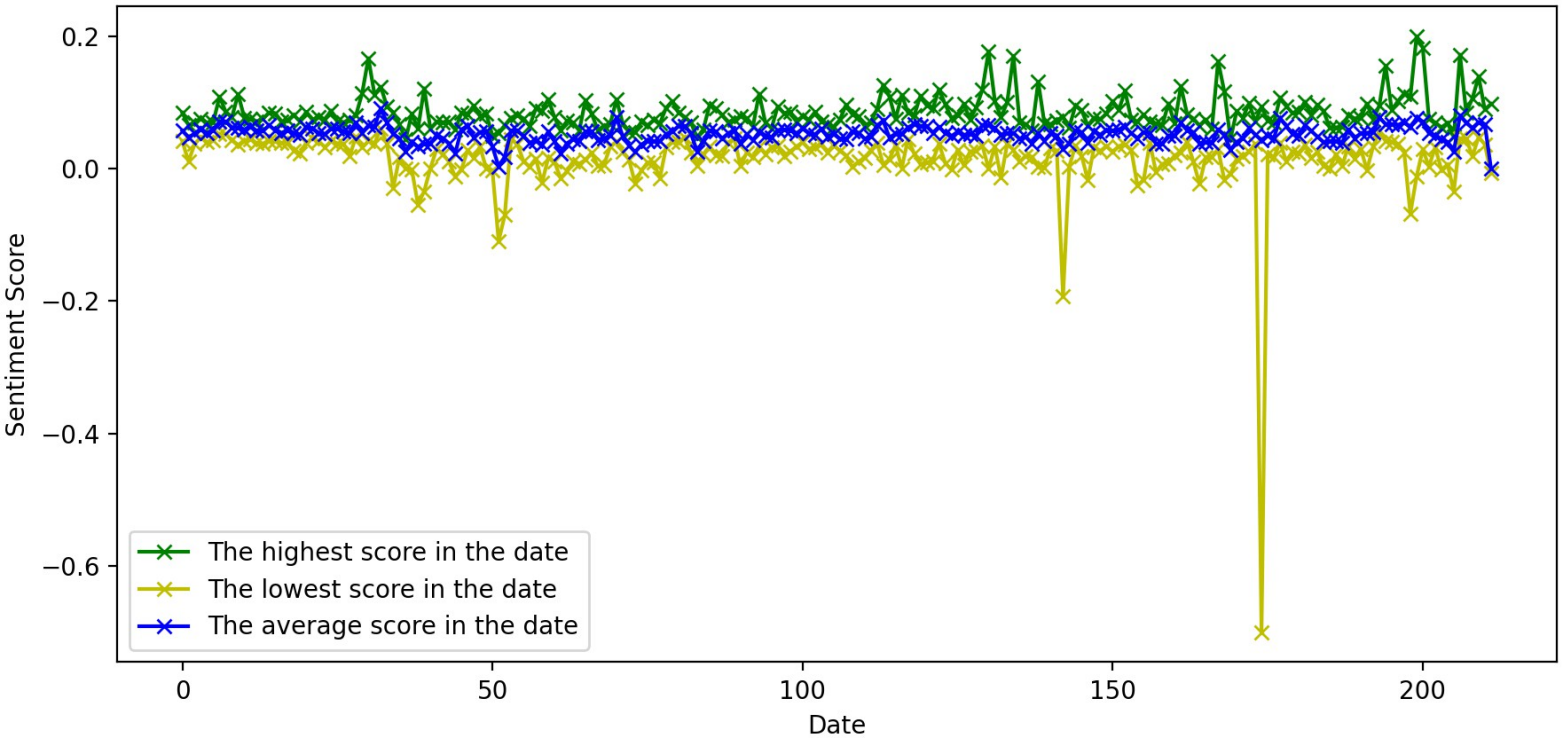
Sentiment score for COVID-19 tweets.

**Fig 2:**
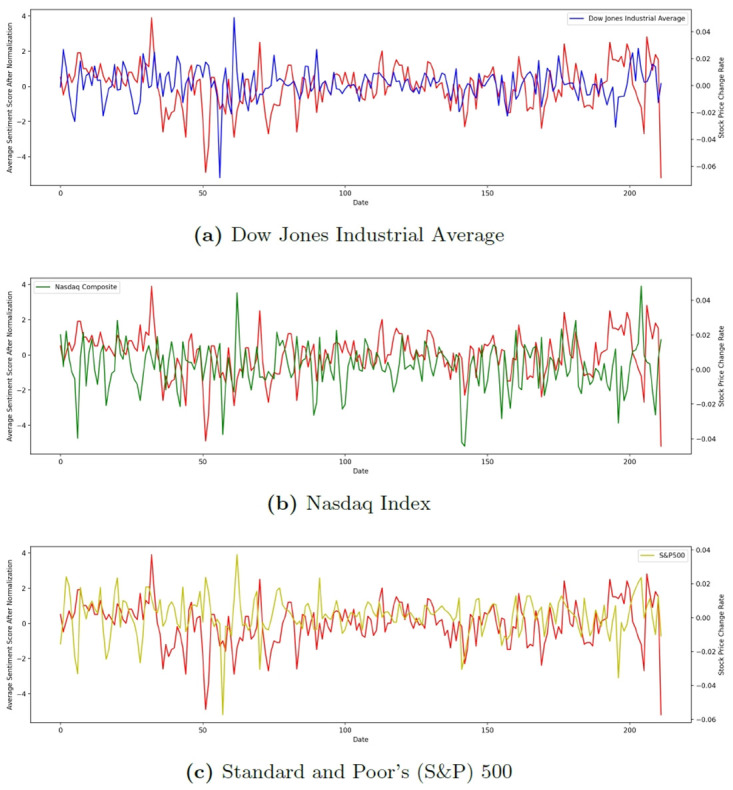
Comparison of fluctuations in the stock index and regularized sentiment scores during COVID-19.

In the sentiment analysis, we found that based on our pre-trained PyTorch sentiment analysis model, the average sentiment score for 98.1% of the dates was greater than 0 (positive) as shown in Fig 1, which could be due to the fact that the majority of Twitter users have a positive view of the COVID-19 situation, or due to the fact that the models used in our analysis were trained on regular Twitter datasets (Sentiment140 [44] and preprocessed-twitter-tweets [45]) generated before pandemic, which may lead the model to exhibit a higher likelihood of positive rather than negative outcomes in the pandemic analysis. When tweeting about COVID-19, the results were somewhat on the positive side. Based on this perception, and to justify the study, we normalized the obtained sentiment scores by $S_{i}=S_{i}-\bar{S}$ and compared them to the three major stock indices, and the results are shown in Fig 2.

#### 3.1.4 Analysis

Based on a comparison of sentiment scores and stock market changes, we can perform a preliminary analysis on the effect of Twitter-related changes in COVID-19 sentiment on stock prices.

The Dow Jones Industrial Average has often been a key indicator of the overall market performance for the U.S. stock market. As the oldest stock price index in the world with a history of over 100 years, it is comprised of only 30 constituent corporations, of which, are the 30 largest and most well-known listed companies in the United States. However, with more than 10,000 stocks listed on the U.S. stock market, many experts and scholars doubt the capability of the DJIA to be an effective market index, with their 30 constituents. Still, we should note that the 30 constituents are all significant corporations in the United States, each with a large reference value that could be used as an indicator of the overall market performance for an investors reference. We can also find from the generated sentiment analysis that the Dow Jones is the one that is most closely correlated with the change in sentiment scores.

The NASDAQ index was created in 1971 as a key indicator of technology stocks around the world. The constituents include all shares listed on the NASDAQ in the United States and is a key indicator of technology stocks around the world. The NASDAQ index has more than 5,000 constituents, covering all aspects of biotechnology, such as computer hardware, software, semiconductors, network communications, etc. It is the preferred reference for investing in technology stocks. But relatively, the changes in the index of technology stocks relative to the changes in sentiment scores relatively large differences, which may be because technology stocks and the company’s own technology level and product technology research and development more relevant, less affected by the COVID-19 situation of public sentiment.

The S&P 500 Index is an overall measure of the top 500 publicly traded companies in the U.S. The rating company Standard & Poor’s has selected 500 leading companies in various industries (and the 500 largest companies in the U.S. with the highest market capitalization) in the U.S. stock market based on market capitalization and liquidity, selected to cover the two major U.S. stock exchanges (New York Stock Exchange and Nasdaq Stock Exchange). The S&P 500 contains more companies than the Dow Jones Industrial Average, and therefore better reflects changes in the stock market and is more risk diversified. In addition, the S&P 500 and the Dow Jones Industrials use different weightings, with the Dow being weighted by stock price and the S&P 500 being weighted by market capitalization, which better reflects the actual value of a company’s stock and can even reflect the rise and fall of the U.S. economy.

### 3.2 JP-Morgan stock sentiments on twitter (2020-2021)

As the COVID pandemic stabilizes, an increasing number of researchers are delving into the impact of the pandemic on markets and stocks. Particularly, some professional researchers in finance have begun to examine how the sentiment expressed on social media affects the stock market. In 2022, researchers at JP-Morgan released a new dataset, TweetFinSent dataset [48]. This is a high-quality, expert-annotated dataset designed for Twitter stock sentiment analysis, focusing on tweets about meme stocks from various companies. It has been rigorously validated against multiple baselines to ensure reliability. This dataset provides a crucial resource for advancing research in stock sentiment analysis by filling the gap of a labeled dataset in this domain.

We retrieved this dataset from a GitHub repository and applied our proposed framework to compute the average sentiment score for each stock on each date. We then aggregated the adjusted closing prices for each stock within the dataset through our framework to create the new dataset. The dataset is employed to assess our proposed S-LSTM model and to demonstrate the effectiveness of our framework in mining market relationships from professional finance social media sentiment.

### 3.3 Long term pandemic sentiment data (2021-2023)

We also aim to examine and record sentiment shifts over the long term of the pandemic using our proposed framework. To this end, we found tweet sentiment data from January 1, 2021, to June 16, 2023, encompassing 661 stock market days. Our framework facilitated the aggregation of adjusted closing prices for selected COVID-related stocks (such as TSLA, ZM, BABA) that we monitor. This method allowed us to create a long-term pandemic sentiment stock dataset to investigate how changes in sentiment during the pandemic have impacted social and market dynamics over time. This dataset was crucial for testing our proposed S-LSTM model as well as demonstrating our findings in the long-term pandemic.

## 4 The proposed method

In 2020, one of the most important events in the world is COVID-19, a global epidemic that has affected all industries and caused extreme volatility in stock market prices. Due to COVID-19 and its impact on work life, we believe that starting with people’s sentiment towards COVID-19 is an excellent attempt to analyze stock prices by exposing the impact of the public’s sentiment towards the pandemic on social media with their investments and information on the financial sector in such an unprecedented pandemic. After the COVID-19 outbreak, many researchers turned their attention to social media and tried to uncover useful information related to COVID-19. Nowadays, Twitter is considered one of the reliable indicators for analyzing the spread of epidemics, and the data generated by users’ activities on social media is becoming one of the important bases for discovering ways to track and analyze epidemic outbreaks [49]. Thus, we use both time series stock index and COVID-19 tweets sentiment analysis score as input data for the proposed S-LSTM model.

**Fig 3:**
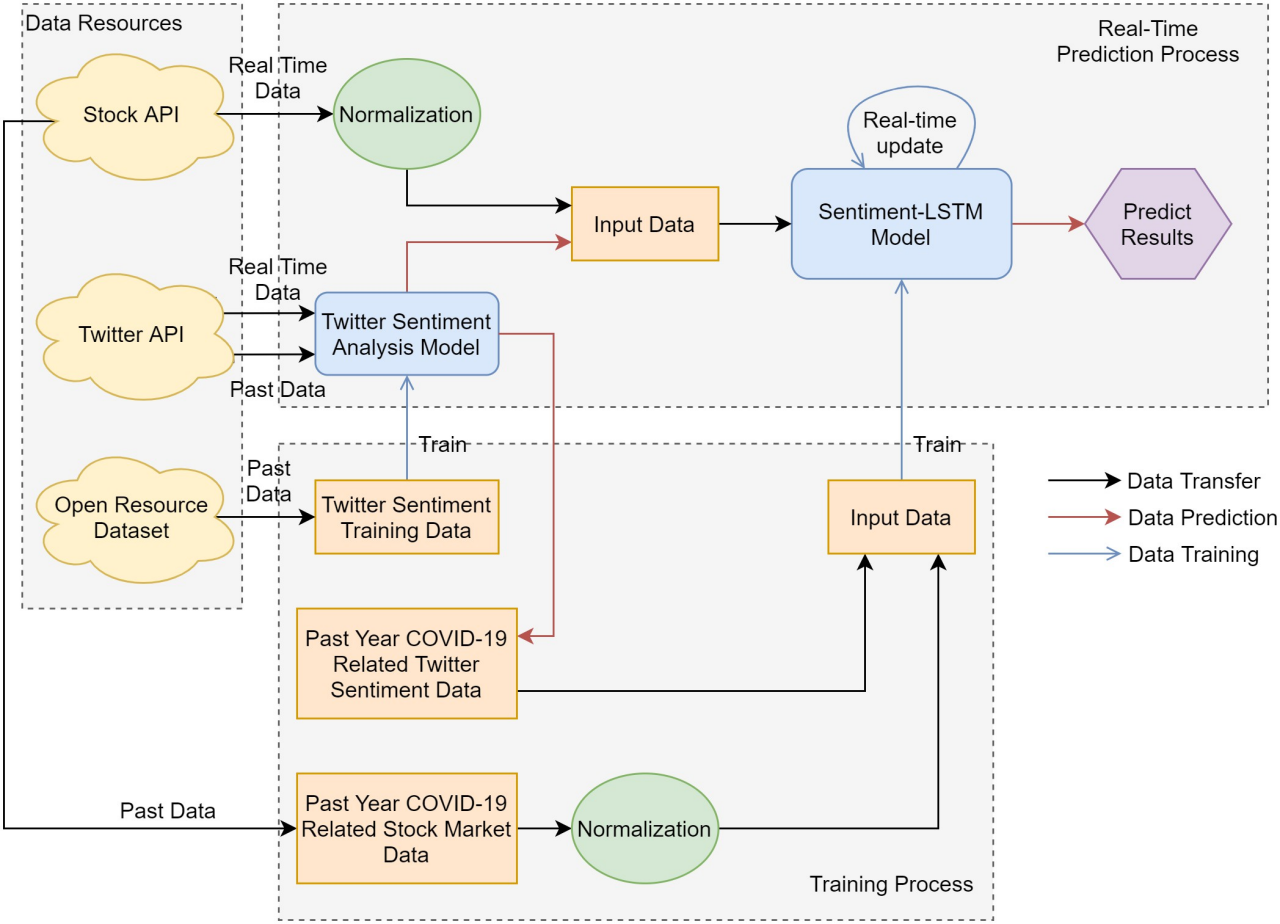
The fundamental process flowchart of the proposed framework.

The fundamental process flowchart of the proposed framework is shown in Fig 3. The whole process can be divided into two major parts, which are Real-Time Prediction Process and Training Process. For the Real-Time Prediction Process, the data resources are Stock API and Twitter API, which can output real-time data for our proposed framework. The Stock API data is normalized as mentioned in Section 3.1.3 and combined with the Twitter Sentiment Analysis Model to be used as input data for the S-LSTM Model. For Training Process, the proposed framework uses an Open Resources Dataset to get Twitter sentiment training data then train the Twitter Sentiment Analysis Model. The S-LSTM Model in our proposed framework can self-update with the real-time data. It can help the proposed model fit the current situation better than a normal LSTM model. Additionally, our proposed framework can preprocess and aggregate sentiment data from various social media sources along with stock data. This data is then used to train the proposed S-LSTM model, enabling us to explore the relationship between these variables.

### 4.1 Time series model

In this paper, we use traditional financial time series of neural networks [50] and the information propagation formula can be written as the following from Eqs (2) to (7):

$$\begin{array}{ccc} f_{t} & = & \sigma (W_{if}\left(x_{t},s_{t}\right)+b_{if}+W_{hf}h_{i-1}+b_{hf}) \end{array}$$ (2)

$$\begin{array}{ccc} i_{t} & = & \sigma (W_{ii}\left(x_{t},s_{t}\right)+b_{ii}+W_{hi}h_{t-1}+b_{hi}) \end{array}$$ (3)

$$\begin{array}{ccc} o_{t} & = & \sigma (W_{io}\left(x_{t},s_{t}\right)+b_{io}+W_{ho}h_{t-1}+b_{ho}) \end{array}$$ (4)

$$\begin{array}{ccc} g_{t} & = & \text{tanh}(W_{ig}\left(x_{t},s_{t}\right)+b_{ig}+W_{hg}h_{t-1}+b_{hg}) \end{array}$$ (5)

$$\begin{array}{ccc} c_{t} & = & f_{t}⊙c_{t-1}+i_{t}⊙g_{t} \end{array}$$ (6)

$$\begin{array}{ccc} h_{t} & = & o_{t}⊙\text{tanh}c_{t} \end{array}$$ (7)

where *f*, *i*, *o* represents the proportionality coefficients of forgetting, input, and output, respectively, and *g*, *c*, *h* represents the candidate state, cell state, and hidden layer state, respectively. The scale coefficients are all used with a sigmoid function to limit the range of coefficients, and the candidate state is related to the information of the input and the hidden state of the previous time layer. The cell state can be considered as a kind of memory cell, and when updating the memory cell, the previous memory is selected to be partially forgotten and the new information is partially accepted, and the hidden layer values get information directly from the current memory cell state into a valve output (the output coefficient *o*_*t*_).

### 4.2 Detail architecture of S-LSTM model

**Fig 4:**
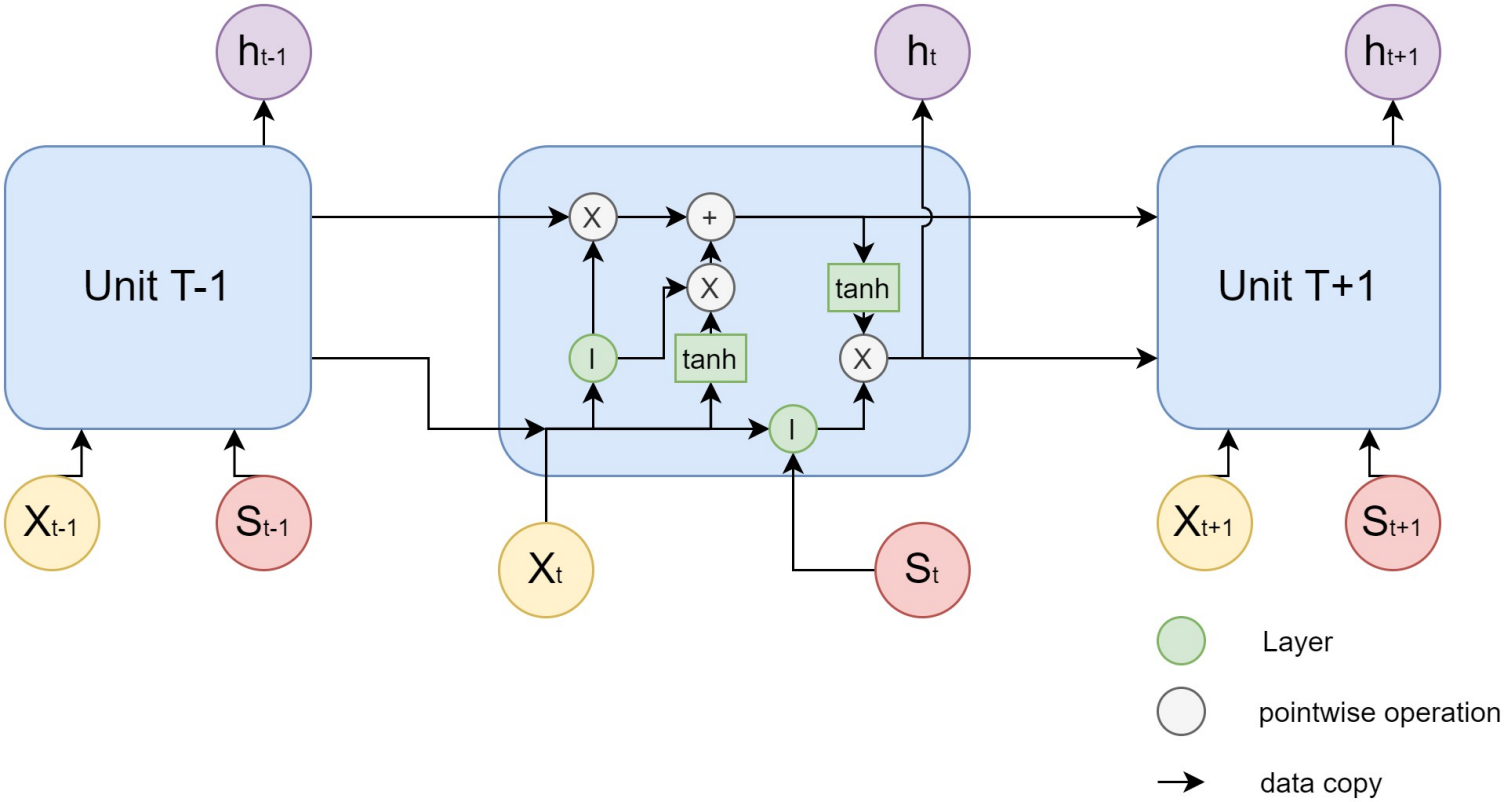
The diagram of Unit T in the proposed S-LSTM algorithm.

Based on the standard time-series LSTM model, we designed the S-LSTM model. This model incorporates the Sentiment Score we generated using the sentiment analysis model as the input data and combines the historical prices of stocks as another part of the input data. The specific algorithm unit can be seen in Fig 4. For each unit, in addition to reading the inputs *X*_*t*_ and *S*_*t*_ at the time point, the results generated from the previous time series are read from Unit *T*−1 as the input at this time point. Unlike traditional LSTM, we read the matrix of the Sentiment Score together with the time points of the algorithm unit and extend the input layers to absorb both stock prices and Sentiment Score and perform learning and regression.

In the diagram, each line transmits an entire vector, from the output of one node to the input of the other nodes. The gray circles represent pointwise operations, such as the sum of vectors, while the green matrix is the learned neural network layer. Lines that are joined together indicate the connection of vectors, and lines that are separated indicate that the content is copied and distributed to different locations.

**Fig 5:**
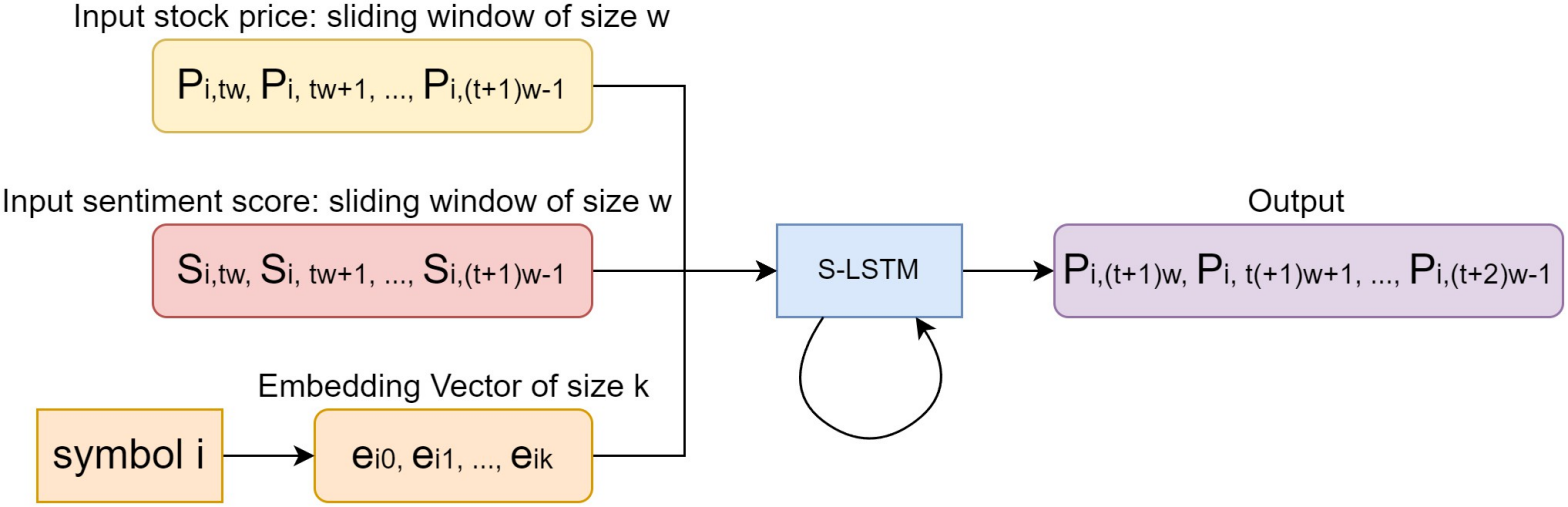
The proposed sentiment-LSTM stock performance mining framework (*w* = *Size*_*input*_, *k* = *Size*_*embedding*_, and symbol *i* can be any stock or stock index).

The Fig 5 explains in more detail how our model handles the data with input and output matrix. From the figure we can see that the input matrix consists mainly of two matrices, stock price and sentiment score at time *t*. The output is the stock price at time *t* + 1.

## 5 Experiment

All the testing algorithms and proposed algorithm are implemented on PowerEdge C4130 Server with 2× Intel Xeon E5-2690 v4 2.6GHz, 35M Cache, 9.6GT/s 1 QPI, Turbo, HT, 14C/28T, 512GB RAM, 4× NVIDIA Tesla P100 16GB Passive GPU. The platform is PyTorch 1.4 and Tensorflow 2.0, the CUDA version is 10.1. Our code is published at GitHub repo: https://github.com/anchensun/S-LSTM.

### 5.1 Real-time tweet sentiment monitoring with market performance

The pre-trained models are used to predict the benchmarks of the stock price changes during COVID-19. We use partial data before the September-October 2020 U.S. election as a test set to avoid the dramatic impact of other major events on stock prices. The Table 2 shows the performance of the major stock market forecasting models on stock price forecasts for the COVID-19 period. As expected, existing deep neural network stock market prediction models are unable to effectively predict stock market prices when major events occur. This is because these prediction models are trained based on data from the past decades and are fitted based on the patterns of those decades and cannot cope with sudden major events or changes. We can find that under normal conditions, some existing prediction models such as TCN, CNN, and LSTM can obtain an accuracy rate greater than 50% and an F1-Score higher than 0.5. However, when testing the trained stock market prediction models with the data during the epidemic, only the LSTM obtained slightly higher than 50% accuracy and none of the tested models could obtain an F1-Score higher than 0.5 for the epidemic test data. Also, every tested prediction model showed more than a 4% drop in accuracy when predicting the test data during COVID-19.

**Table 2:** Performance of the main forecasting models in the DJIA data set.

Model	Accuracy(Normal)	F1-Score(Normal)	Accuracy(Covid-19)	F1-Score(Covid-19)	Difference
ARIMA	48.16%	0.345	43.40%	0.298	-4.76%
TCN	56.48%	0.514	48.69%	0.441	-7.79%
CNN	53.28%	0.522	49.13%	0.473	-4.15%
LSTM	55.71%	0.508	51.21%	0.451	-4.50%

The algorithm we use to uncover the relationship between the stock market and social media sentiment is Sentiment(S)-LSTM, which, as mentioned before, can combine past stock market data to extract time-series patterns and combine them with the social media sentiment scores we obtain from our sentiment analysis model to learn and obtain a stock market index prediction model. The input data types for the different tested model are shown in Table 3, where 1) Time series stock price data R: stock price dataset consisting of daily records of the Dow Jones Industrial Average; 2) Text news data N: news dataset consisting of historical news from the Reddit WorldNews channel; 3) Twitter sentiment analysis data S: sentiment scores generated based on relevant Twitter data.

**Table 3:** Input data for different models.

Model	ARIMA	CNN	TCN	WB-TCN	LSTM	S-LSTM
Raw Data	R	R	R	N	R	S + R
Processed Training Data	R	R	R	word embedding	R	sentiment score + R

**Fig 6:**
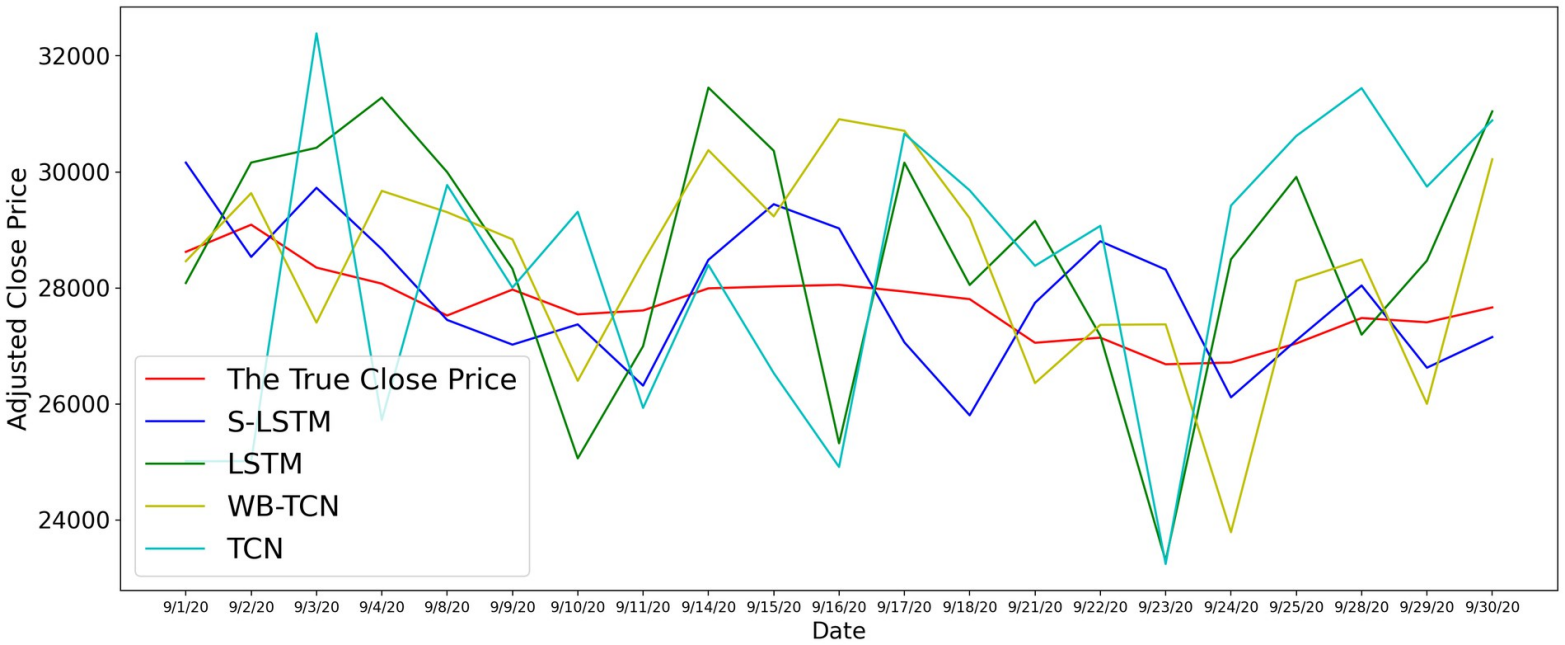
Sep 2020 stock Dow Jones Industrial Index close price prediction comparison.

We tested different models using the Dow Jones Industrial Index Close Prize for each day of September 2020 as a test dataset. The input data are the Dow Jones Industrial Index Close Prize for the previous three days, the Twitter sentiment scores for the previous three days, and the COVID-19 related news text data for the previous three days. This is due to the relative stability of the U.S. stock market in September 2020, as well as the absence of events of great impact both domestically and internationally in the United States. Compared to November, the U.S. stock market is more volatile due to the election, which is not conducive to testing the performance of different models relative to COVID-19. The results of the test are shown in Fig 6.

We use equation $|\frac{P_{predict}-P_{truth}}{P_{truth}}|$ to calculate the accuracy of different tested models and shown in Table 4, the high percentage means that the model can obtain the correct trend (up or down) when predicting the stock price at the next point in time. In this test, we can clearly see that S-LSTM is the most accurate prediction algorithm, with a significant improvement compared to the traditional LSTM. Meanwhile, WB-TCN, which combines news text output, also shows advantages over traditional TCN and LSTM. This proves that the model combining more outputs has unparalleled advantages in dealing with unexpected events.

**Table 4:** Input data for different models.

Model	TCN	WB-TCN	LSTM	S-LSTM
Accuracy	53.37%	63.74%	60.01%	**70.52**%

In order to compare the performance of the proposed algorithm for different single stock price predictions, we selected three stocks from different industries that are highly affected by COVID-19, AMC, CCL, and PFE, for our experiments. Like other companies in the industry, AMC Theatres has been negatively impacted by the coronavirus pandemic. As a result of the outbreak, AMC Theatres was forced to close hundreds of theaters. Upon reopening, they experienced low customer traffic as customers preferred to stay home to watch movies over watching movies at the theatres. Hollywood blockbusters were delayed due to COVID-19 as well. In October 2020, AMC Theatres warned investors that its dwindling cash reserves could force it to file for bankruptcy protection. Before the outbreak, the company was already $4.75 billion in debt. Carnival Cruise Lines (CCL) is one of the top three cruise lines in the world. As of now, there is a lot of uncertainty about the cruise industry’s return to normal operations. All three of these major cruise lines ended their 2020 fiscal year with record losses due to the New Crown Pneumonia outbreak. Carnival Corporation reported a net loss of $10.236 billion on revenues of $5.595 billion. These results reflect the enormous impact of the New Crown Pneumonia outbreak on the entire cruise industry. Pfizer (PFE) was the first major pharmaceutical company to announce the effectiveness of its new crown vaccine. So far this year, Pfizer’s stock price has fallen before Moderna Inc. (MRNA) took a hit in November 2020 by announcing the effectiveness of its competing vaccine. In 2020, pharmaceutical stocks under-performed the rest of the class in the U.S. stock market, not an obvious outcome against the backdrop of a new crown epidemic spurring drug development. Even biotech stocks, of which Moderna is a part, have under-performed the S&P 500.

The training data is the closing price of these three stocks during February 2020 to July 2020. The testing data is the closing price of these three stocks in August 2020 and September 2020. The models will use the closing stock price and sentiment score of the previous days as inputs to predict the closing stock price of the following day. We calculate the performance (lower means better performance) of the proposed S-LSTM algorithm by Eqs (8) and (9):

$$\begin{array}{ccc} Diff_{t} & = & \frac{|P_{true,t}-P_{prediction,t}|}{P_{true,t}} \end{array}$$ (8)

$$\begin{array}{ccc} Performance_{stock} & = & \sqrt{\frac{\sum_{t=1}^{n}Diff_{t}^{2}}{n-1}} \end{array}$$ (9)

and the results are shown in Table 5. We can find that among the three stocks, the performance of our proposed S-LSTM is consistently the best. In comparison, PFE achieves relatively good performance on this stock for all four algorithms because of its small variation. For the two stocks, AMC and CCL, investors are more sensitive to the changes of the epidemic, so our proposed S-LSTM algorithm and the WB-TCN algorithm, when used as a comparison, appear to be more accurate for price prediction due to the combination of crowd sentiment scores for COVID-19 on social media or keyword extraction for COVID-19’s news vocabulary. Specifically, our proposed algorithm incorporating sentiment scores provides better prediction results than previous WB-TCNs.

**Table 5:** Performance of stock closing price prediction results for the four algorithms for the three stocks during August and September 2020.

Stock	S-LSTM	LSTM	WB-TCN	TCN
AMC	**0.11472**	0.24194	0.17614	0.25891
CCL	**0.12040**	0.23028	0.173659	0.26957
PFE	**0.11525**	0.17657	0.14922	0.21379

**Fig 7:**
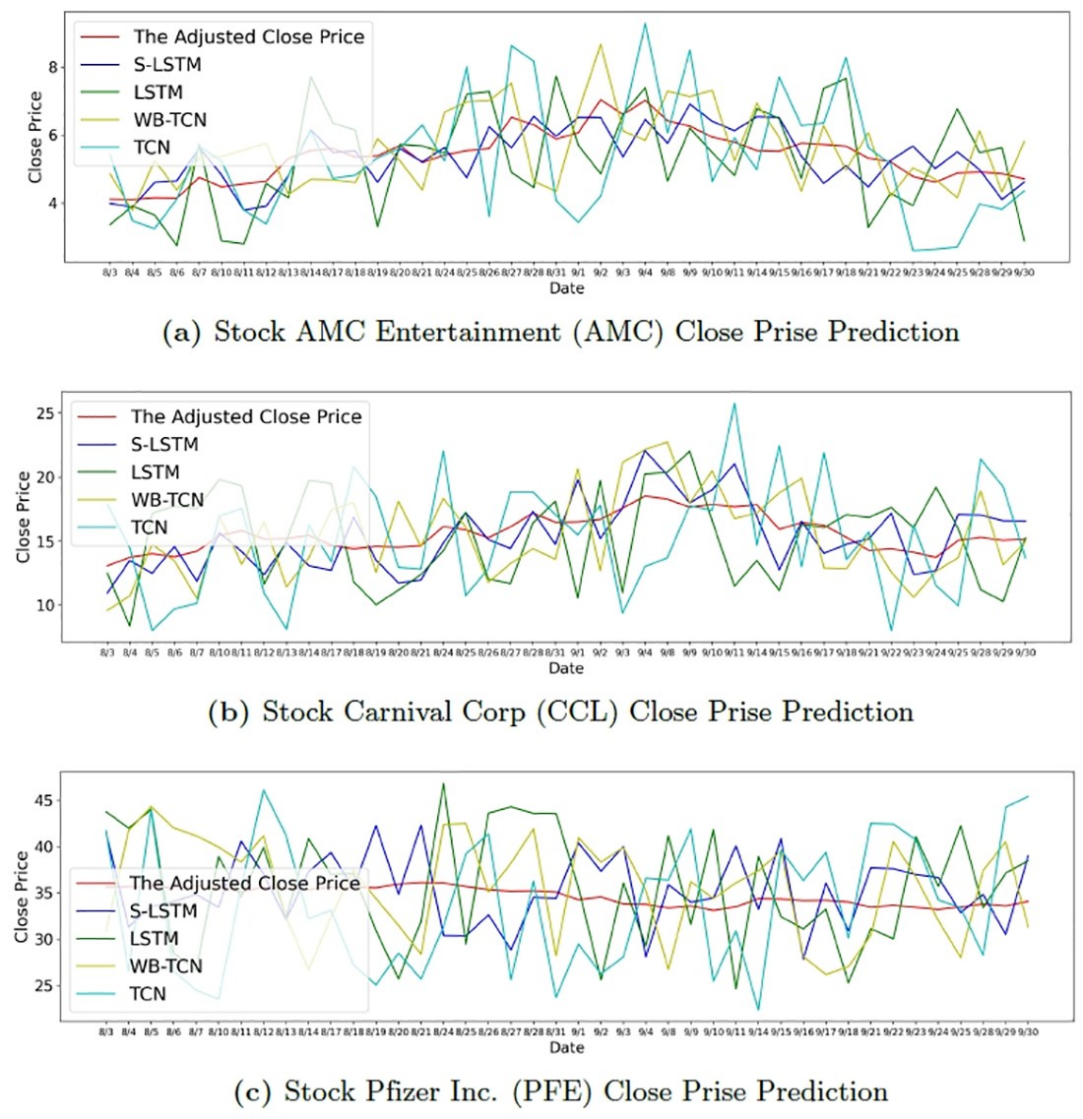
August and September 2020 stock close price prediction comparison.

We also plot out the forecast results to show in the Fig 7, and we can see that among the three stocks, PFE stock has relatively the least change. All algorithms on PFE zoom in on the changes and appear to be not very accurate. CCL and AMC are more sensitive to changes in the COVID-19 outbreak due to the large changes and the properties of the stocks themselves, so the closing prices of the stocks predicted by our proposed algorithm are more in line with the actual trend.

### 5.2 TweetFinSent for specific stock performance

After completing data cleaning and aggregating stock adjusted close prices, we have a dataset comprising 228 days of training data encompassing 15 different stocks. Among these, Tesla (TSLA) appears on 174 dates, AMC on 122 dates, GameStop (GME) on 96 dates, and so forth, spanning from September 1, 2020, to August 31, 2021. Additionally, our testing dataset comprises 226 days and includes 18 different stocks. For instance, Palantir Technologies (PLTR) is represented on 103 dates, Alibaba Group (BABA) on 102 dates, Clover Health Investments (CLOV) on 80 dates, Zoom Video Communications (ZM) on 75 dates, and others. The testing data spans the same timeframe as the training data, from September 1, 2020, to August 31, 2021.

**Fig 8:**
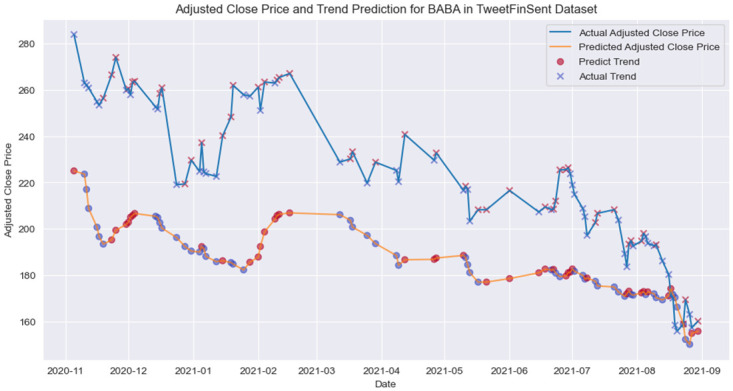
Adjusted close price and trend prediction for BABA in TweetFinSent dataset.

We use 6 previous days as input sequence, and the experimental results are shown in Table 6, and we plot the prediction versus actual adjusted close price for BABA with trend detection in Fig 8. Due to a shortage of professional finance news data for word embedding, our testing was limited to the S-LSTM, LSTM, and TCN models. Our findings indicate that the S-LSTM model outperformed the others across all four test stocks. This validates the relationship between professionally labeled finance social media sentiment data and actual stock market performance.

**Table 6:** Accuracy of adjusted stock closing price prediction results for the four algorithms for the four stocks of TweetFinSent dataset.

Stock	S-LSTM	LSTM	TCN
CLOV	**0.67538**	0.59892	0.62185
PLTR	**0.55856**	0.51503	0.54553
BABA	**0.63534**	0.56847	0.61649
ZM	**0.66427**	0.62832	0.63631

### 5.3 2021 to 2023 country level sentiment dataset

We applied our proposed framework to gather and compute the adjusted closing prices for six COVID-related stocks and two stock indices (Nasdaq and S&P 500). We selected one stock for testing and used the remaining stocks and indices as training data to train and evaluate our proposed S-LSTM model using 6 previous days as the input sequence.

We then calculated the accuracy for the years 2021, 2022, and 2023, with the results presented in the Table 7. In our analysis, we observed that stocks such as AMC, CLOV, PLTR, and ZM demonstrated high accuracy in trend prediction for the year 2022 compared to 2021 and 2023, suggesting a strong correlation with tweet sentiment during 2022. This could reflect a resurgence in the American market in 2022, with increased participation from citizens in the stock market, paralleled by a heightened expression of sentiment on social media. However, in 2023, the correlation between social media sentiment and the stock market appeared to diminish, indicating a stabilization or calming of market activities. In contrast, Tesla (TSLA) exhibited consistent prediction accuracy across all three years, underscoring a potential stability in the relationship between social media sentiment and TSLA’s stock performance, with minimal fluctuations. Another noteworthy observation is Alibaba (BABA), which displayed strong prediction accuracy in 2023. This may be attributed to increased optimism in China regarding the end of the pandemic and a renewed engagement with the global market, drawing greater attention to BABA among investors and the general public.

**Table 7:** Accuracy of adjusted stock closing price prediction results for the S-LSTM for the six stocks of Long-term 2021 to 2023 Tweet sentiment dataset.

S-LSTM	2021	2022	2023	Overall
TSLA	63.21	62.20	61.00	62.17
AMC	61.44	65.42	56.36	62.15
CLOV	59.38	64.62	55.45	60.99
PLTR	56.50	63.41	50.00	58.34
BABA	61.85	61.41	71.82	63.64
ZM	55.68	59.00	55.45	56.85

## 6 Discussion

As we have shown in Table 2, LSTM neural network may not be enough to predict stock market price. It is combined with text sentiment over a short period of time in our experiment, as seen in Section 4, that we can make full use of this model. Based on the results, our implicates that when sentiment is applied to neural networks, most notably the S-LSTM model, it will yield better results and can be used as a model for adjusted closing price of stocks to a high degree of accuracy.

Quantitative analysis work can be divided into three parts from a broad perspective: macro, meso and micro. Macro, through the study of macroeconomic data to analyze and judge the future economic trends, meso is based on industry data, industry trends, rotation, etc., while micro is based on the company’s fundamental data, stock selection.

Economic trends will largely be reflected in the stock market, the economic form is good, the stock market for the probability of bull market. When the economic fundamentals are weak and downward pressure is high, the stock market is lukewarm. For example, in 2018, the overall economic downturn, the stock market fell more, and stock market trends and macroeconomic trends show a strong correlation. Macroeconomic forecasts will be the most important reference when making investment decisions, and industry and firm-level analysis will be done on this basis.

The performance of various models on a test set of stock market data during COVID-19 highlighted significant shortcomings in predicting complex stock market dynamics, particularly during major unforeseen events. A more comprehensive prediction model that incorporates additional information, such as news or social media data, can offer more reliable forecasts.

Specifically, incorporating the emotional responses from Twitter users regarding COVID-19 significantly enhanced the predictive accuracy of the LSTM model. Compared to the text output from TCN for news, Twitter sentiment analysis using streaming tweet data showed greater improvements.

Furthermore, we utilized the TweetFinSent dataset and long-term tweet sentiment scores from 2021 to 2023 to validate our proposed framework. This demonstrated that the S-LSTM model is effective not only with real-time social media data but can also integrate other data sources to consolidate market information and explore the relationship between social media sentiment analysis and the stock market. This approach underscores the potential of our framework for professional asset management and economic research.

## 7 Conclusion

In conclusion, this research sought to explore the potential of leveraging text sentiment from social media as a predictive tool for stock market prices. By utilizing social media as an alternative news source, we aimed to enhance econometric methodologies for understanding and forecasting market trends. The S-LSTM model emerged as the most accurate tool in our study, demonstrating the effectiveness of advanced data processing techniques for both text sentiment analysis and financial time series data concerning market index performances.

Throughout our analysis, we tailored specific fits for each index and assessed the performance of various some popular models, with real-time streaming tweet sentiment analysis conducted in late 2020. The S-LSTM model consistently outperformed other methods, highlighting its robustness and reliability.

Further, we utilized a professionally labeled finance tweet sentiment dataset spanning from late 2020 to late 2021 to illustrate how the S-LSTM model can effectively integrate and collaborate with other data sources, thus reinforcing its superior performance. Our extended research from 2021 to 2023 demonstrated the framework’s ability to learn and mine long-term data, effectively revealing trends and assisting researchers in understanding the prolonged impact of social media sentiment on the market. This long-term analysis is crucial for developing more nuanced and comprehensive strategies in financial market analysis.

Following recent advancements in large language models, there is potential to integrate these models into our framework for more detailed information extraction from social media, beyond simply generating sentiment scores. Additionally, incorporating a broader range of sentiment features from multiple sources, or utilizing multimodal data encompassing environmental, social, and governance (ESG) factors, and then aggregating this with stock market data through our framework, represents another promising research direction [51]. Such approaches could offer more comprehensive insights and significantly enhance decision-making in management practices.
